# Long-term efficacy and safety of steroid-eluting sinonasal stents in endoscopic dacryocystorhinostomy: a prospective, single-arm study

**DOI:** 10.3389/fsurg.2026.1916619

**Published:** 2026-08-05

**Authors:** Mu Xian, Yifan Meng, Menglin Wang, Yi Zhou, Kuiji Wang, Xiaohong Song, Yi Dong, Guoqiang Zhao, Ying Jie, Chengshuo Wang

**Affiliations:** 1 Department of Otolaryngology Head and Neck Surgery, Beijing TongRen Hospital, Capital Medical University, Beijing, China; 2 Research Ward, Beijing TongRen Hospital, Capital Medical University, Beijing, China; 3 Beijing Key Laboratory of Nasal Diseases, Beijing Institute of Otolaryngology, Beijing, China; 4 Beijing Tongren Eye Center, Beijing Institute of Ophthalmology, Beijing Tongren Hospital, Capital Medical University, Beijing, China

**Keywords:** chronic dacryocystitis, efficacy, endoscopic dacryocystorhinostomy, nasolacrimal duct, safety, steroid-eluting stents

## Abstract

**Background:**

Chronic dacryocystitis resulting from lacrimal pathway obstruction is a persistent condition associated with bothersome symptoms.

**Objective:**

This study aimed to evaluate the long-term efficacy and safety of steroid-eluting sinus stents in endoscopic dacryocystorhinostomy (EN-DCR).

**Methods:**

This study enrolled patients who underwent EN-DCR between July 2021 and April 2022. Adult patients with chronic dacryocystitis caused by primary lacrimal duct obstruction were enrolled. After completion of the conventional EN-DCR procedure, a steroid-eluting sinus stent designed for use in the frontal sinus was implanted at the ostium. Patients were followed up for 54 months after surgery. Operative time, stent removal time, and symptoms at each follow-up visit were recorded. The endoscopic ostium patency rate and lacrimal irrigation success rate were analyzed using the Kaplan–Meier product-limit survival analysis.

**Results:**

A total of 31 patients (33 eyes) were included. At 12, 45 and 54 months, the neo-ostial patency rates were 96.97% (95% confidence interval [CI] 80.37%–99.57%), 93.94% (95% CI 77.88%–98.45%) and 90.70% (95% CI 73.84%–96.91%), with 3 eyes experiencing ostial occlusion over 54 months. Lacrimal irrigation success rates matched patency rates at 12 and 45 months (96.97% and 93.94%, identical 95% CIs), declining to 84.22% (95% CI 66.12%–93.12%) at 54 months in 5 eyes with irrigation failure. Serum cortisol concentrations stayed within normal range, with no stents-related adverse events documented postoperatively.

**Conclusions:**

Implantation of steroid-eluting sinonasal stents during EN-DCR achieves favorable long-term safety and efficacy by preserving neo-ostial patency. Nevertheless, dedicated stents anatomically tailored to the neo-ostium of EN-DCR are required to further optimize surgical outcomes.

## Introduction

Chronic dacryocystitis is a common inflammatory or infectious condition of the lacrimal sac that typically results from persistent obstruction at the junction of the lacrimal sac and the nasolacrimal duct (1). It affects approximately 1%–3% of the adults worldwide and accounts for about 3% of ophthalmology clinic visits (1, 2). Epiphora and purulent discharge are the most common presenting symptoms (2). This condition significantly impairs quality of life and imposes substantial social and economic burdens (3).

Currently, two primary surgical approaches are used to treat nasolacrimal duct obstruction: endoscopic dacryocystorhinostomy (EN-DCR) and external dacryocystorhinostomy (EX-DCR). Although earlier studies reported no significant differences in functional and anatomical outcomes between EX-DCR and EN-DCR after an average follow-up of 8 months (4), EN-DCR has become the preferred first-line intervention because of its shorter operative time, fewer complications, and higher success rate (5, 6). The reported success rate of EN-DCR is approximately 85% across various studies (6, 7). However, outcomes remain suboptimal, as some cases fail due to granulation tissue formation and scarring at the neo-ostium (1, 8). Persistent inflammation around the neo-ostium and exposed bone lacking nasal mucosal coverage are considered key contributing factors. This limitation is particularly concerning, as evidence suggests that revision EN-DCR may provide sustained benefit for up to 2 years (5). Accordingly, various strategies have been explored to prevent neo-ostium synechiae. Techniques such as silicone stenting, polypropylene tubes, bicanalicular silicone intubation, mitomycin-C application, and mucosal flap suturing techniques have shown efficacy (1, 9). However, some studies suggest that implanted materials may themselves promote granulation tissue formation near the neo-ostium (1, 10), whereas others report that mucosal flap preservation may not be essential for long-term success (11). Thus, there is a need for a novel device that provides structural support, sustained anti-inflammatory effects, and biodegradability to prevent synechiae.

Steroid-eluting stents are innovative devices introduced into clinical practice in 2011 for localized corticosteroid delivery (12). Composed of biodegradable polymers and impregnated with corticosteroids, these stents gradually degrade after implantation, releasing a concentrated dose of corticosteroids directly to affected mucosal tissues. Randomized controlled trials (RCTs) have demonstrated their efficacy and safety following endoscopic sinus surgery in patients with chronic rhinosinusitis (13). Notably, placement of biodegradable steroid-eluting stents in the frontal sinus ostium significantly reduces scarring and inflammation, maintains ostial patency, and improves surgical outcomes such as in Draf III (14). The stent also helps to fix the mucosal flap, preventing its dislodgement and displacement. Given the anatomical similarities between the nasolacrimal duct ostium and frontal sinus ostium—both characterized by narrow channels and small openings—we hypothesized that a steroid-eluting stent designed for the frontal sinus could be effectively applied in EN-DCR. This approach may provide both mechanical support and sustained anti-inflammatory effects, thereby reducing neo-ostium synechiae and improving long-term outcomes.

This study aimed to evaluate the long-term efficacy and safety of steroid-eluting sinus stents in EN-DCR. To our knowledge, this is the first clinical investigation to assess the use of steroid-eluting stent in EN-DCR with a focus on long-term outcomes.

## Materials and methods

### Study design and participants

This research was designed as a prospective single-center, single-arm trial, conducted in the Department of Rhinology at Beijing Tongren hospital, China. Adult patients with chronic dacryocystitis secondary to primary lacrimal duct obstruction who underwent EN-DCR between July 2021 and April 2022 were enrolled. Exclusion criteria included congenital obstructions, dacryocystitis secondary to trauma, granulomatous diseases (e.g., Wegener's granulomatosis or sarcoidosis), prior radiotherapy, history of external DCR, revision EN-DCR, eyelid anatomical abnormalities, and impaired lacrimal pump function.

This study was approved by the Ethics Committee of Beijing Tongren hospital and was conducted in accordance with Good Clinical Practice guidelines and the Declaration of Helsinki. All patients were provided with detailed study information and instructions, and written informed consent was obtained before enrollment. This study was designed, conducted, and reported in accordance with the Strengthening the Reporting of Observational Studies in Epidemiology (STROBE) Statement. The completed STROBE checklist is provided as Supplementary File S1.

### Surgical procedure

**Figure 1:**
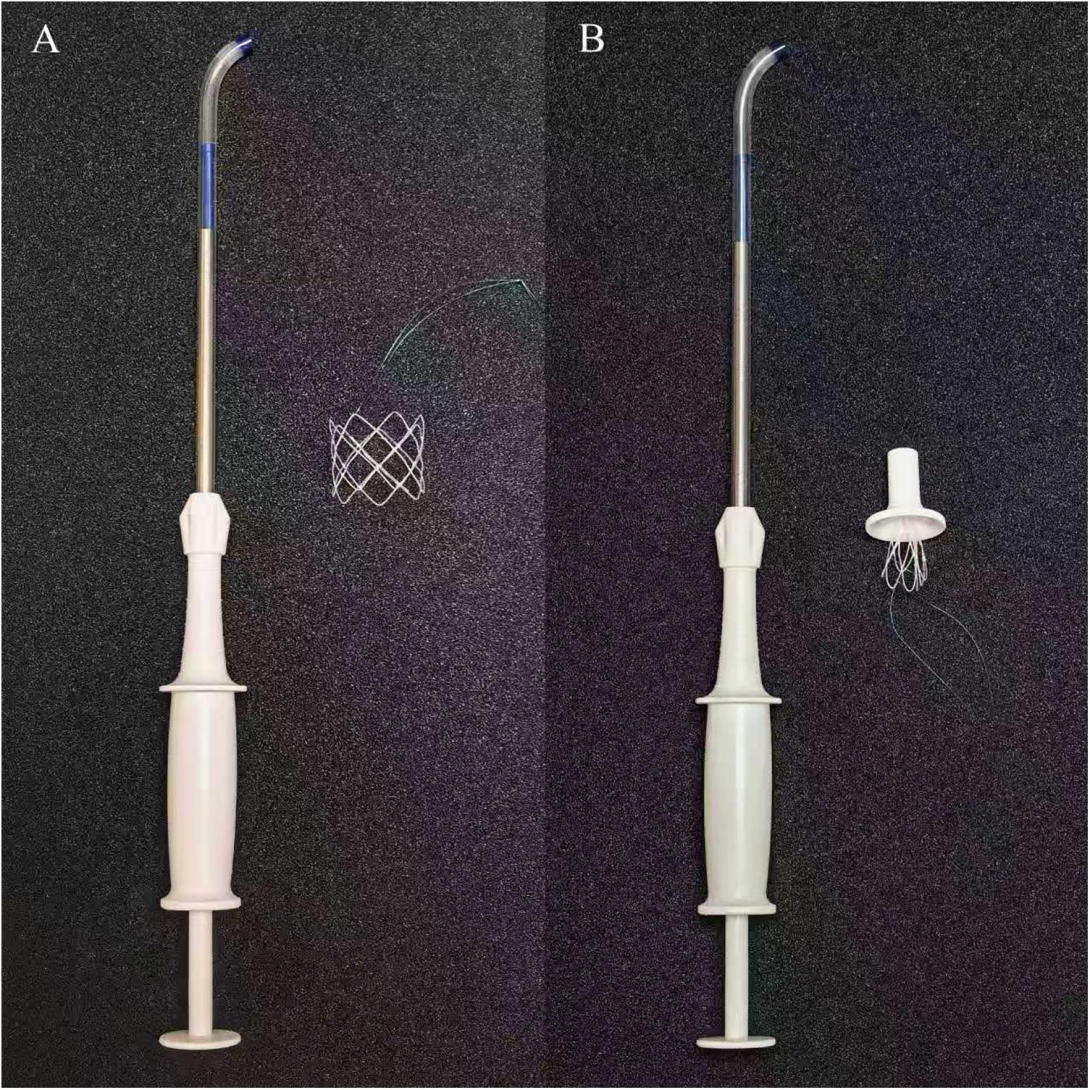
Steroid-eluting stent designed for the frontal sinus and its delivery device. **(A)** The steroid-eluting stent and delivery device, featuring a curved tip, are designed to match the angle of the frontal sinus drainage pathway. **(B)** The steroid-eluting stent was placed within the cap for deployment at the tip of the device.

**Figure 2:**
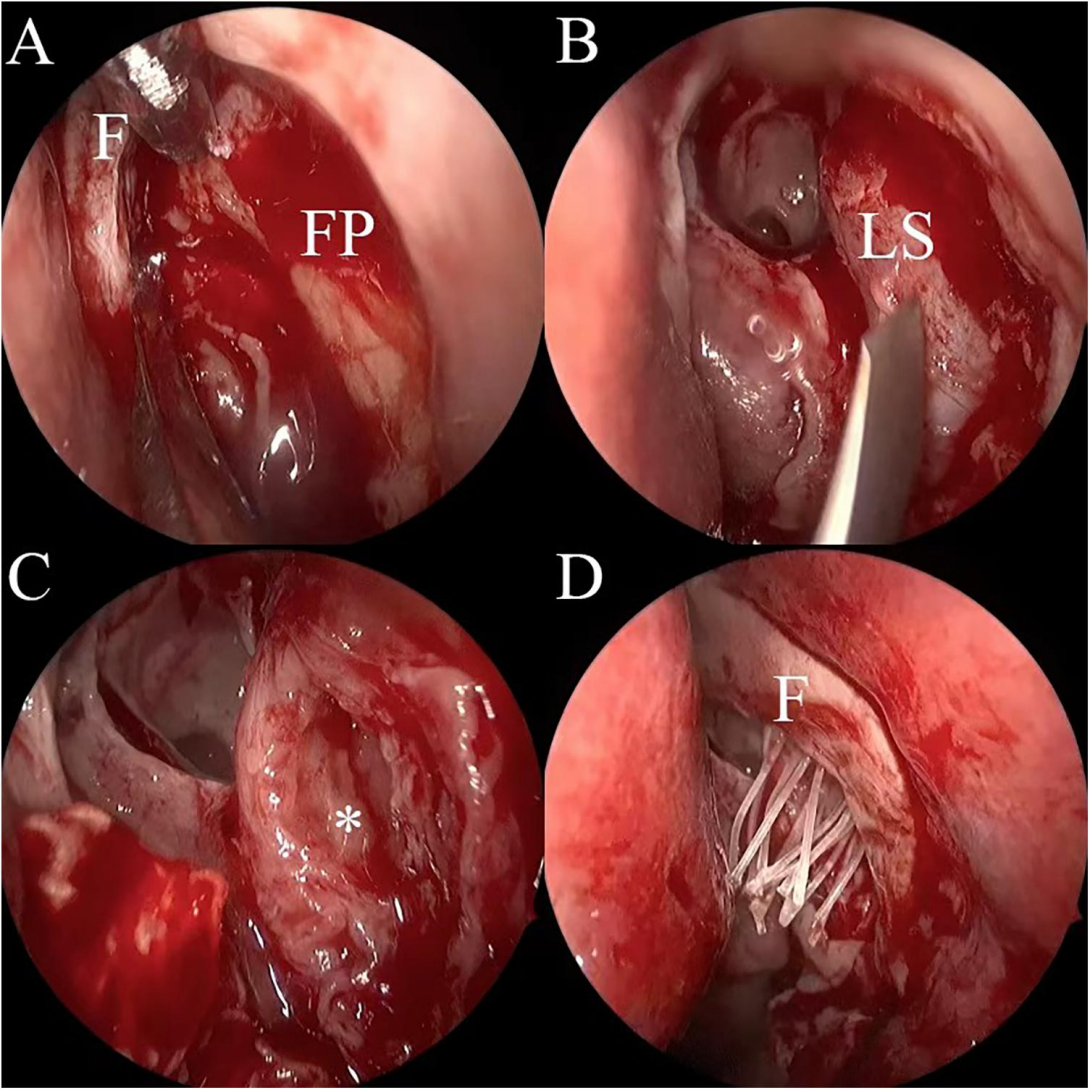
Application of the frontal sinus steroid-eluting stent in endoscopic dacryocystorhinostomy. **(A)** The mucosal flap over the left frontal process was elevated superiorly to expose the frontal process of the maxilla. **(B)** The maxillary frontal process was drilled to expose the lacrimal sac. **(C)** The lacrimal sac was incised and opened, with the lumen identified (white asterisk, *). **(D)** A steroid-eluting stent was implanted after thorough irrigation of the lacrimal drainage system, and the mucosal flap was subsequently repositioned to cover the denuded bone. F, mucosal flap; FP, frontal process of the maxillary; LS, lacrimal sac.

All patients underwent preoperative computed tomography (CT) dacryocystography to assess lacrimal sac dimensions. All EN-DCR procedures were completed under general anesthesia by experienced rhinologists. During EN-DCR, a mucosal flap was elevated superior to the medial lacrimal eminence to expose the underlying bone. The lacrimal bone was elevated posteriorly and inferiorly using a Freer elevator. The maxillary process was dissected using either a Kerrison punch or a microdrill to fully expose the lacrimal sac (15). The sac was incised in a “]”-shaped fashion and reflected posteriorly to allow apposition to the nasal mucosa. Either the inferior or superior punctum was dilated and irrigated with saline. Steroid-eluting sinus stents originally designed for frontal sinus (Xiangtong; Puyi Biotechnology, Shanghai, China), composed of polylactide-co-glycolide polymers and coated with mometasone furoate, were trimmed to fit the neo-ostium and inserted using a specialized introducer (Figure 1). The mucosal flap was then repositioned (Figure 2).

### Postoperative care and follow-up schedule

Postoperatively, patients received oral antibiotics (amoxicillin-clavulanate) for 5–7 days. Nasal and lacrimal irrigation was initiated 2 days after surgery, along with a corticosteroid nasal spray. Topical levofloxacin and fluorometholone eye drops were administered three times daily and discontinued for 4 weeks. Follow-up visits were scheduled at 1, 2, 4, 8, 12 weeks, 45 months, and 54months postoperatively. Stents were removed at 1, 2, or 4 weeks based on the recovery status of the nasal mucosa surrounding the neo-ostium according to the investigators' judgement.

### Study outcomes

Baseline demographic and clinical data including age, sex, presence of comorbid nasal disease, lacrimal sac size (defined as the maximum product of the anteroposterior diameter and lateral diameters of the lacrimal sac on horizontal CT images), laterality (unilateral or bilateral), operative time, stent removal time, symptoms of epiphora, endoscopic findings of the DCR neo-ostium patency, lacrimal irrigation success rate, and adverse events were recorded at each follow-up visits. Serum cortisol concentrations were measured at postoperative day 2 and the 12-week follow-up visit. Anatomical success is defined as a patent neo-ostium confirmed by endoscopy. Functional success is defined as successful lacrimal irrigation together with resolution of epiphora without revision surgery.

### Statistical analysis

Given its exploratory pilot nature and the absence of prior long-term clinical data regarding steroid-eluting stents in EN-DCR, no *a priori* power analysis was performed to calculate sample size. A total of 30 subjects were prospectively enrolled. All statistical analyses were performed using GraphPad Prism (Version version 11, GraphPad Software, San Diego, CA, USA). The normality of continuous variables was tested prior to analysis. Continuous data were expressed as mean ± standard deviation (SD). Categorical variables were summarized as counts and percentages (*n*, %).

Kaplan–Meier product-limit survival analysis was performed to estimate the ostium patency rate and lacrimal irrigation success rate over 54 months of follow-up. Event was defined as ostium occlusion for ostium patency outcome and failed lacrimal irrigation for irrigation success outcome, respectively. All eyes without an endpoint event at the end of 54-month follow-up and those lost to follow-up at 45 months were treated as right-censored observations. The 95% confidence intervals (CIs) for survival probabilities at predefined time points (12, 45, and 54 months) were calculated using the log-log transformation based on Greenwood's variance estimator to constrain CI bounds within the range of 0 to 1.

## Results

### Baseline patient characteristics

A total of 31 patients (33 eyes) were screened. Of these, 31 patients (33 eyes) who met the inclusion and exclusion criteria were enrolled. Among them, 30 patients (32 eyes) completed the 54-month follow-up, while 1 patient (1 eye) was lost to follow-up at month 54. The mean ± SD age of patients was 57.5 ± 13.5 years, with 8 (25.8%) male and 23 (74.2%) female. The anteroposterior diameter and lateral diameters on maximum cross-sectional area of the lacrimal sac on preoperative axial CT were 3.8 ± 0.3 and 5.1 ± 0.4 mm, respectively. Four patients (4 eyes) had a history of prior tube intubation. Common comorbidities included nasal septal deviation (2 patients), rhinosinusitis (2 patients), and nasal tumors (1 patient). Necessary concomitant procedures, such as endoscopic septoplasty, nasal tumor dissection, and sinusotomy, were performed during the EN-DCR.

### Intraoperative and follow-up outcomes

**Figure 3:**
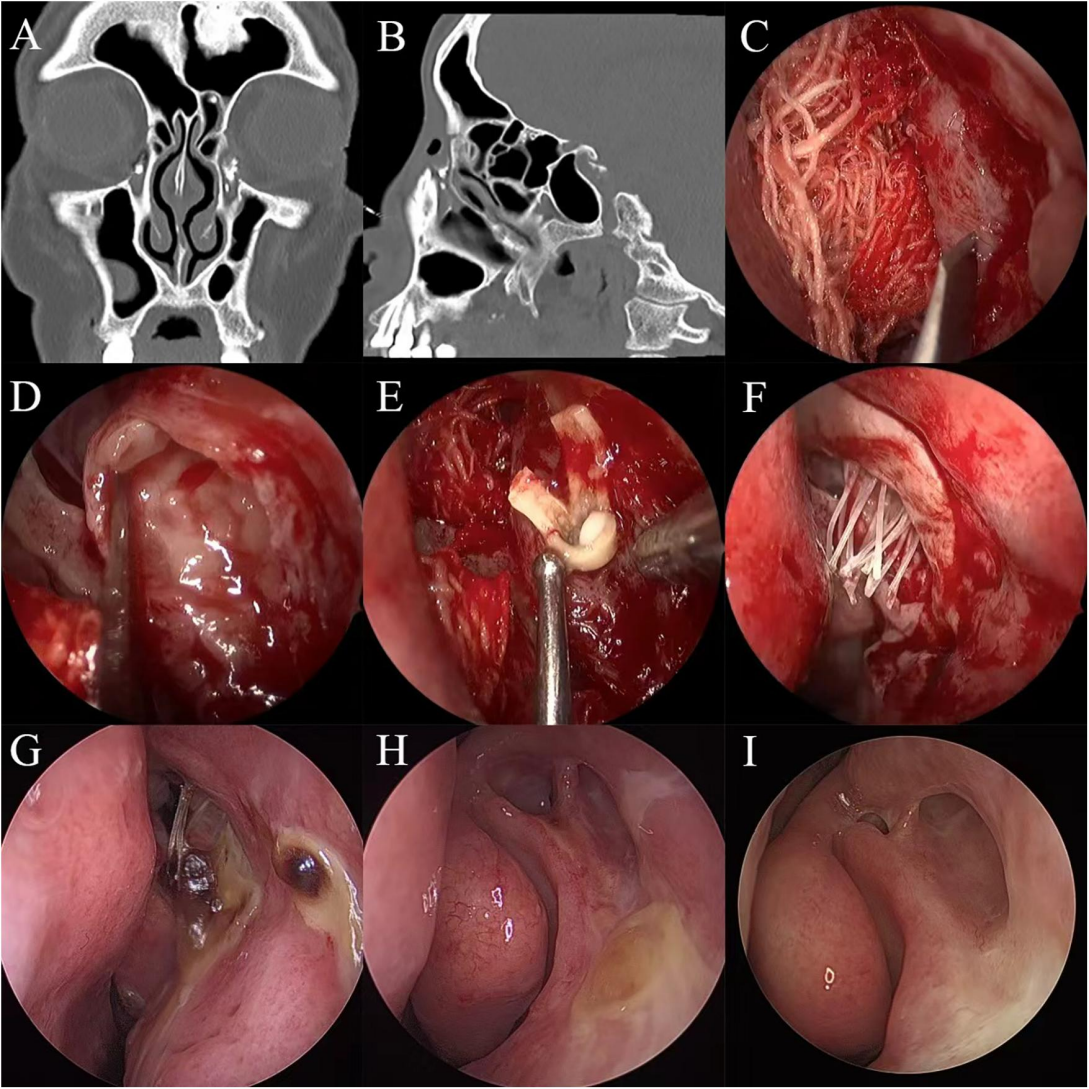
Representative case demonstrating the application of a steroid-eluting stent in endoscopic dacryocystorhinostomy (EN-DCR). **(A,B)** Coronal and sagittal computed tomography (CT) demonstrating left lacrimal passage obstruction and a previously implanted silicone intubation that has remained in the lacrimal passage long-term. **(C)** The maxillary frontal process was drilled to expose the lacrimal sac. **(D)** Edematous lacrimal sac mucosa. **(E)** Residual silicone tube identified in the lacrimal sac. **(F)** A steroid-eluting stent was implanted. **(G)** Endoscopic view at 2 weeks post-EN-DCR showing the stent with minor crusting around the neo-ostium. **(H)** Endoscopic view at 4 weeks post-EN-DCR showing a patent lacrimal sac lumen with the stent having been removed. **(I)** Endoscopic view at 12 months postoperatively showing a patent lacrimal sac lumen.

The mean operative time was 70 ± 8.7 min. Seventeen stents were removed 1 week after EN-DCR, 12 stents were removed 2 weeks after EN-DCR and 4 were removed 4 weeks after EN-DCR. Figure 3 illustrates the key steps of implanting a trimmed steroid-eluting frontal sinus stent at the neo-ostium following left-sided EN-DCR.

**Figure 4:**
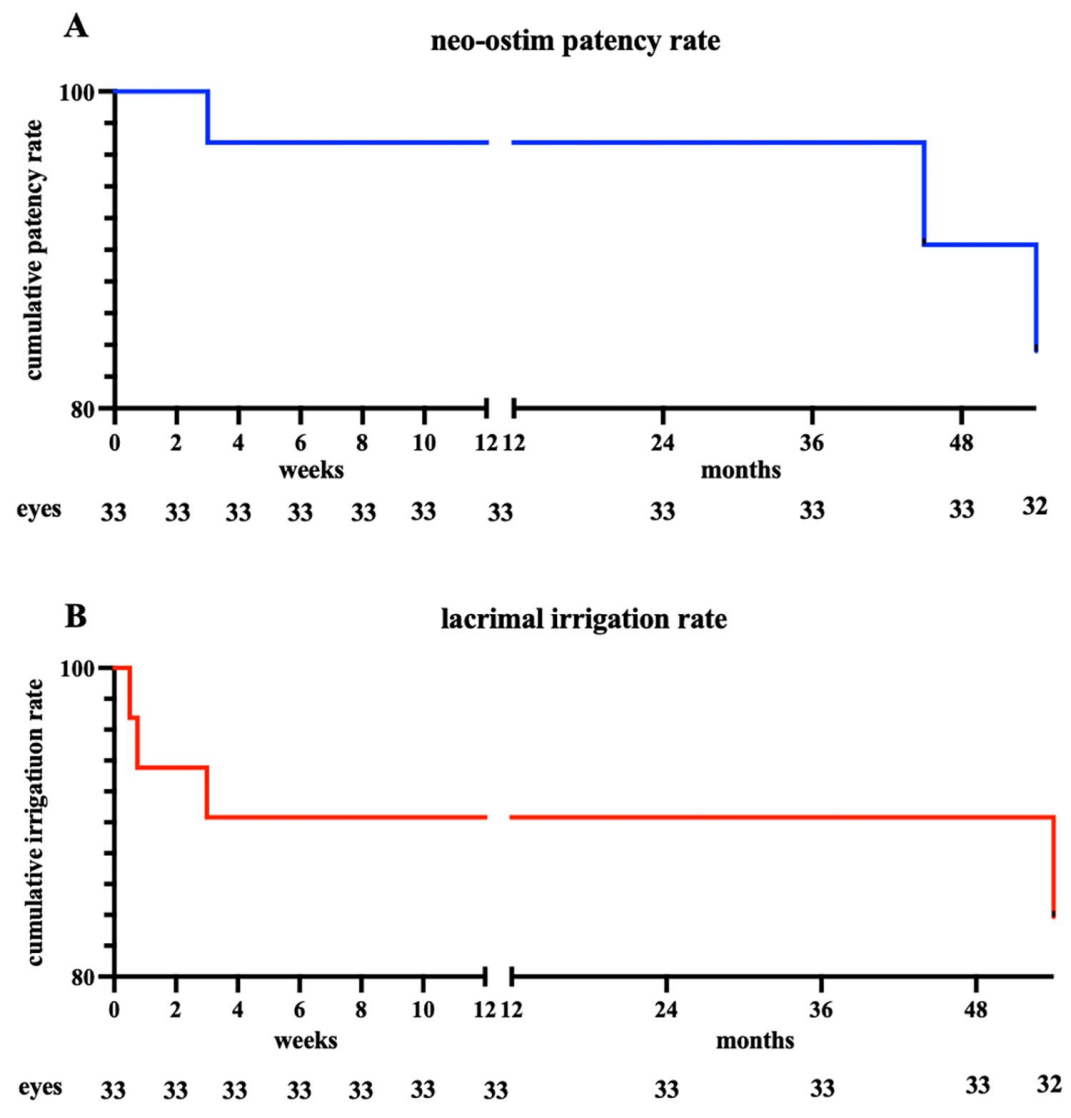
Kaplan–Meier survival curves for anatomical and functional success following steroid-eluting stent-assisted endoscopic dacryocystorhinostomy over 54 months of follow-up. **(A)** Kaplan–Meier curve estimating neo-ostium patency rates. **(B)** Kaplan–Meier curve estimating lacrimal irrigation success rates.

A total of 33 eyes were included for survival analysis of both ostium patency and lacrimal irrigation outcomes. For ostium patency rate, the estimated patency probabilities at 12, 45, and 54 months were 96.97% (95% CI: 80.37%–99.57%), 93.94% (95% CI: 77.88%–98.45%), and 90.70% (95% CI: 73.84%–96.91%), with 3 eyes developing ostium occlusion during the entire follow-up period (Figure 4A). For lacrimal irrigation success rate, the survival curves overlapped with those of ostium patency up to 45 months, yielding identical success rates of 96.97% (95% CI: 80.37%–99.57%) at 12 months and 93.94% (95% CI: 77.88%–98.45%) at 45 months; the 54-month lacrimal irrigation success rate decreased to 84.22% (95% CI: 66.12%–93.12%), corresponding to 5 eyes with persistent irrigation failure at the final follow-up timepoint (Figure 4B). Notably, the lacrimal irrigation success rate was consistent with subjective symptomatic findings documented in medical records. No median failure time was reached within the 54-month observation window for either endpoint.

### Safety and adverse event profiles

Serum cortisol concentrations (reference range: 5–23 μg/dL) stayed within the physiological normal range at postoperative day 2 (15.7 ± 5.1 μg/dL) and the 12-week follow-up visit (16.6 ± 7.2 μg/dL), with no steroid-eluting sinonasal stents-related adverse events documented throughout the postoperative follow-up period.

## Discussion

In this prospective, single-arm study, we evaluated the long-term efficacy and safety of steroid-eluting sinonasal stents in EN-DCR. Our findings demonstrate that this approach is both safe and effective, yielding high rates of anatomical and functional success over a 54-month follow-up period. Specifically, the estimated neo-ostial patency rate remained at 90.70%, and the lacrimal irrigation success rate was 84.22% at the 54-month mark. These results suggest that steroid-eluting stents are a promising option for improving long-term surgical outcomes in patients with nasolacrimal duct obstruction.

Nasolacrimal duct obstruction is the most common underlying cause of chronic dacryocystitis, and surgical intervention has become the preferred treatment modality. EN-DCR, first introduced in 1989, was rapidly adopted because of its shorter operative time, lower complication rate, and reduced morbidity (16). However, neo-ostium synechiae remain a major challenge largely due to persistent inflammation at the neo-ostium, as well as granulation tissue formation and scarring (1, 8). Although several studies have demonstrated satisfactory short-term outcomes, long-term efficacy remains suboptimal (6, 16–18). Consequently, numerous studies have focused on reducing surgical failure rates and prolonging the time to recurrence (5, 6, 9, 11, 16–20). Accordingly, this study aimed to investigate a novel implant and evaluate its long-term efficacy and safety in the management of this condition.

In the present study, we applied a sinus steroid-eluting stent in EN-DCR. The stent provides mechanical support and stabilizes the lacrimal mucosal flap, preventing stenosis and atresia of the neo-ostium secondary to postoperative displacement of mucosal flaps, which to some extent reduces the difficulty of surgery. Previous studies have demonstrated that steroid-eluting stents reduce polyp recurrence, decrease mucosal inflammatory burden, and improve olfaction (13, 21). Various types of steroid-eluting sinus stents have been developed for different anatomical sites, including the frontal sinus, ethmoid sinus, maxillary sinus, and sphenoid sinus. Given the frontal sinus drainage tract shares the closest similarity to the lacrimal sac in diameter and angular orientation, a steroid-eluting frontal sinus stent was utilized in the current investigation.

Postoperatively, the stents were removed upon the investigators' judgement that the neo-ostium had formed, the surrounding mucosa had healed, and the steroid-eluting sinonasal stents no longer provided anti-inflammatory or mechanical support. Stents were removed within 2 weeks in 27 patients (29 eyes). In four patients (4 eyes), stent removal was delayed until 4 weeks postoperatively because of persistent crusting around the neo-ostium. Although these stents are designed to be biodegradable, we observed that localized granulation tissue formation at the neo-ostium was more common in patients whose stents were removed at 4 weeks postoperatively. This may be partly attributed to the fact that the frontal sinus stents used in our study had a slightly larger diameter than the lacrimal sac, resulting in greater local tension. Alternatively, it may be due to the histological differences between the lacrimal sac epithelium and the sinonasal mucosa. This pilot study highlights the necessity of designing lacrimal stents that are better tailored for EN-DCR neo-ostia in terms of configuration, mechanical support, and degradation profile.

The success of EN-DCR can be evaluated based on functional or anatomical outcomes. Some studies rely solely on anatomical criteria, whereas others require both criteria to be met (6). In the present study, both outcomes were evaluated, given the relatively small differences reported between these measures (6, 22). At 12 weeks postoperatively, the rates of neo-ostial patency and successful lacrimal irrigation both reached 96.97%. During long-term follow-up, both rates declined slightly; nevertheless, they remained relatively high (90.70% and 84.22% at 54 months, respectively). One patient developed epiphora due to neo-ostium synechiae associated with granulation tissue formation. Another patient developed epiphora with purulent discharge in one eye due to acute dacryocystitis following an upper respiratory infection. Previous studies evaluating stent use in EN-DCR, in which outcomes were defined based on anatomical or functional success, have reported success rates of approximately 78% at 12 months and 86% at 5 years after surgery, although follow-up durations and stent types varied (6, 19). This study represents the first pilot investigation to utilize a sinus steroid-eluting stent in EN-DCR. None of the four patients with prior lacrimal tube intubation developed recurrent epiphora throughout the 54-month follow-up period, suggesting that steroid-eluting sinonasal stents may confer therapeutic benefits to patients with refractory or recurrent lacrimal obstructive disease (Figure 3).

This study has several limitations. First, this investigation was designed as a prospective pilot, single-arm study to evaluate feasibility, long-term safety, and clinical performance rather than superiority over conventional EN-DCR. Due to variations in patient inclusion criteria, operating surgeons, and follow-up durations, the results of this study cannot be directly compared numerically with the postoperative recurrence rates of traditional DCR reported in previous studies. Second, several potential sources of selection bias should be acknowledged. Patient recruitment was limited to a single tertiary referral rhinology center, and all EN-DCR procedures were performed by experienced rhinologists. In addition, stringent exclusion criteria were applied to exclude patients with congenital, traumatic, inflammatory granulomatous, or revision lacrimal disorders, which narrows the spectrum of participants included in this study. Third, only adult participants were recruited in this study. Pediatric patients possess unique anatomical features, wound healing profiles, and surgical management considerations (23). Given the inherently narrower nasolacrimal ducts in children, stenting may deliver superior therapeutic outcomes in this population. Therefore, future prospective trials with customized stents are warranted for pediatric patients.

Several factors may restrict the direct extrapolation of our results to other clinical institutions, while the core biological mechanism reported in this study retains broad theoretical generalizability. First, all participants were recruited exclusively from a single high-volume tertiary referral rhinology center specializing in lacrimal surgery. Such standardized, high-expertise operative conditions are not universally available. Second, the strict predefined inclusion and exclusion criteria reduce selection heterogeneity within our cohort but inevitably limits the ability to extend our exact success rates to unfiltered real-world patient populations seen at general hospitals. Third, the stent used in this investigation is a modified frontal sinus steroid-eluting stent rather than a device anatomically customized for EN-DCR neo-ostia. Variations in stent size trimming protocols, and local stent supply across different hospitals may alter clinical outcomes in external cohorts. Nevertheless, the core mechanistic principle, that steroid-eluting stents sustain neo-ostial patency through mechanical support of the mucosal flap combined with local anti-inflammatory drug release, possesses biological generalizability.

## Conclusion

Steroid-eluting sinonasal stents constitute a safe and effective strategy for preserving neo-ostial patency after EN-DCR. Moreover, this stent technique demonstrates promising efficacy in refractory patients with prior tube intubation. However, compared to the trimmed frontal sinus stents used in this study, there is a clear need to design lacrimal stents specifically tailored for EN-DCR. To our knowledge, this is the first study documenting the long-term efficacy of steroid-eluting sinus stents in EN-DCR. These findings lay the foundation for the development of specialized lacrimal stents, the implementation of prospective multicenter comparative trials against traditional DCR, and further investigations focusing on refractory cases and pediatric populations.

## Data Availability

The raw data supporting the conclusions of this article will be made available by the authors, without undue reservation.
